# *In vitro* Activity of Allicin Alone and in Combination With Antifungal Drugs Against *Microsporum canis* Isolated From Patients With Tinea Capitis

**DOI:** 10.3389/fmed.2021.783086

**Published:** 2021-11-26

**Authors:** Ya Bin Zhou, Yuan Yuan Xiao, Jin Jing Chao, Lin Ma

**Affiliations:** Department of Dermatology, Beijing Children's Hospital, Capital Medical University, National Center for Children's Health, Beijing, China

**Keywords:** *Microsporum canis*, tinea capitis, allicin, drug combination, antifungal drug

## Abstract

The checkerboard broth method based on the Clinical and Laboratory Standards Institute M38-A3 document was used in this study to evaluate the *in vitro* activity of allicin alone and in combination with the antifungal drugs (griseofulvin, fluconazole, itraconazole and terbinafine) against *Microsporum canis* isolated from patients with tinea capitis. When allicin was used alone, only weak anti-*M. canis* effects were found. The MIC_50_, MIC_90_ and geometric mean (GM) of terbinafine were the lowest among the compounds tested. Synergism was observed for the combinations of allicin with itraconazole and terbinafine. Only indifference was observed for the combinations of allicin with griseofulvin and fluconazole. Our study illustrated the synergism of allicin in combination with itraconazole and terbinafine, which could be a reference for the treatment of tinea capitis due to *M. canis*.

## Introduction

Tinea capitis is a common dermatophytosis of the scalp and hair follicles. A previous study on the epidemiology of tinea capitis in children suggested that *Microsporum canis* is the most prevalent pathogen in most areas of China (1).

Griseofulvin, terbinafine, fluconazole and itraconazole are most common used antifungal drugs for tinea capitis. However, resistance to antifungal drugs is emerging in *M. canis* (2). A strain of griseofulvin resistant *M. canis* with a high minimum inhibitory concentration (MIC) was isolated from recalcitrant tinea capitis which had treatment with griseofulvin (3). Recently, a strain of terbinafine resistant *M. canis* was isolated from a cat which failed to topical terbinafine treatment (4). High *in vitro* MICs of azoles were also been noted in *M. canis* (5). To cope with drug-resistant mycoses, a combination of traditional antifungal drugs with non-antifungal agents has been proposed as a promising treatment strategy (6).

Allicin, a sulfur compound from garlic, has been demonstrated to have activity against *Candida, Cryptococcus, Trichophyton, Epidermophyton*, and *Microsporum* (7). Although allicin and fluconazole are synergistic *in vitro* against *Candida albicans* (8), no studies have been conducted on the *in vitro* activity of allicin in combination with antifungal drugs against *M. canis*.

In the present study, the *in vitro* antifungal activity of allicin alone or in combination were assessed against 30 clinical *M. canis* strains. The results will help us evaluate the therapeutic potential of allicin in combination with antifungal drugs against tinea capitis.

## Materials and Methods

### Microorganisms

A total of 30 *M. canis* strains were tested. They were all recovered from the hair of patients with tinea capitis at the Outpatient Clinic of Dermatology (Beijing Children's Hospital, Capital Medical University). Each isolate was stored in 10% glycerol at −80°C. *Candida parapsilosis* (ATCC 22019) and *Trichophyton interdigitale* (ATCC MYA 4439) were chosen as quality controls.

All strains were identified microscopically and with molecular sequencing of the internal transcribed spacer (ITS) region. Genomic DNA of each strain was extracted by using a Biospin Fungus Genomic DNA Extraction kit (Bioer Technology Ltd; Hubei, China) in accordance with the manufacturer's instructions. The extracted DNA was eluted with 100 μl of distilled water, and 1 μl of the extracted DNA was used for polymerase chain reaction (PCR). The ITS region was amplified using the primers ITS1 (5′-TCCGTAGGTGAACCTGCGG-3′) and ITS4 (5′-GGTCCGTGTTTCAAGACGG-3′). Each PCR mixture contained 1 μl of extracted fungal DNA, 0.08 μM each of the primers, and 12.5 μl of 2 × Taq PCR MasterMix (Tiangen Biotech Ltd; Beijing, China) in 25 μl of reaction volume. The PCR cycling comprised an initial denaturation at 95°C for 5 min, followed by 35 cycles of 95°C for 30 s, 58°C for 30 s, and 72°C for 1 min, followed by a final extension at 72°C for 10 min and cooling to 4°C. The amplicon products were then sequenced by Tian Yi Hui Yuan Company (Beijing, China). The sequences obtained were compared with those in the GenBank DNA database (https://blast.ncbi.nlm.nih.gov/). The sequences obtained had been deposited in GenBank with the following accession numbers: MT163398-MT163427.

### Chemicals

All drugs were acquired from Harvey Biotech Ltd., (Beijing, China) and dissolved in dimethyl sulfoxide (DMSO). The stock solution concentrations were as follows: 12.8 mg/mL for allicin; 6.4 mg/mL for fluconazole; 1.6 mg/mL for itraconazole, griseofulvin and terbinafine. The drugs were analyzed in the following final concentration ranges: 2–128 μg/mL for allicin; 0.015–8 μg/mL for itraconazole and griseofulvin; 0.125–64 μg/mL for fluconazole; and 0.001–0.5 μg/mL for terbinafine. The final concentration of DMSO did not exceed 1% in test wells.

### Checkerboard Microdilution Assay

Assays were performed according to the checkerboard broth microdilution method based on the Clinical and Laboratory Standards Institute M38-A3 document (9, 10). Briefly, all strains enrolled in our study grew for 14 days on potato dextrose agar at 28°C, and conidial suspensions were prepared by gently scraping the surfaces of the fungal colonies into sterile physiological saline. Heavy hyphae fragments were settled for 5 min at room temperature and the upper, conidial suspensions were used as an inoculum. Susceptibility tests were then performed in RPMI 1,640 medium (Gibco, USA) supplemented with 0.3 g/l L-glutamine but without sodium bicarbonate and buffered to pH 7.0 with 0.165 M 4-morpholinopropanesulfonic acid (Amresco, USA). The final concentration of the suspension diffused in the wells was adjusted to ~1–3 × 10^3^ CFU/mL, as determined with a hemocytometer. Serial 2-fold dilutions of 50 μl of each drugs A (allicin) and B (fluconazole, itraconazole, griseofulvin or terbinafine) were dispensed along the vertical and horizontal directions to yield 100 μl per well in a 96-well microtiter plate. One hundred microliters of the diluted inoculum suspension was dispensed into each well. The plates were incubated at 35°C for 4–5 days. The MICs were determined as the lowest concentration producing a 100% reduction in turbidity by visual observation with a concave mirror when compared with the drug-free control. All experiments were conducted in replicate on different days.

### Drug Interaction Analysis

The drug combination interaction was evaluated on the basis of the fractional inhibitory concentration index (FICI), which is the sum of the MIC of each drug in combination divided by the MIC of the drug used alone. The drug interaction was defined as follows: FICI ≤ 0.5, synergism; FICI >0.5 and ≤ 4.0, indifference; and FICI >4.0, antagonism (9).

## Results

The results of the *in vitro* susceptibility tests of *M. canis* strains to the antifungal drugs alone are listed in Tables 1, 2. When allicin was used alone, only weak anti-*M. canis* effects were found (MICs, 16–128; GM, 46.313 μg/ml). We also found that the MIC_50_, MIC_90_ and geometric mean (GM) of terbinafine were the lowest. Among the tested azoles, fluconazole had a higher MIC_50_, MIC_90_ and GM than itraconazole.

**Table 1:** *In vitro* interactions of allicin with griseofulvin, fluconazole, itraconazole, and terbinafine against *M. canis* strains.

**Strains**	**MIC (μg/mL)**			**FICI**	**MIC (μg/mL)**		**FICI**	**MIC (μg/mL)**		**FICI**	**MIC (μg/mL)**		**FICI**
	**Allicin**	**Griseofulvin**	**Allicin/griseofulvin**		**Fluconazole**	**Allicin/fluconazole**		**Itraconazole**	**Allicin/itraconazole**		**Terbinafine**	**Allicin/terbinafine**	
BCH32585	32	1	32/1	2	16	32/16	2	1	16/0.25	0.75	0.06	8/0.004	0.313
BCH32598	32	0.5	32/0.5	2	32	16/16	1	0.5	4/0.125	0.375	0.015	4/0.002	0.125
BCH32773	32	1	32/1	2	64	32/64	2	1	4/0.06	0.188	0.015	8/0.004	0.5
BCH32775	128	1	64/0.5	1	32	32/16	0.75	1	32/0.06	0.313	0.03	16/0.004	0.25
BCH32785	64	0.5	32/0.25	1	16	64/16	2	0.5	16/0.03	0.313	0.03	8/0.002	0.188
BCH32820	64	0.5	64/0.5	2	16	64/16	2	1	4/0.25	0.313	0.06	4/0.002	0.099
BCH32823	64	1	64/1	2	64	32/8	0.625	0.25	8/0.015	0.188	0.008	16/0.002	0.5
BCH32860	32	0.25	32/0.25	2	16	32/16	2	0.25	4/0.015	0.188	0.06	4/0.004	0.188
BCH32931	32	1	16/0.5	1	32	32/32	2	0.25	8/0.06	0.5	0.004	16/0.001	0.75
BCH33015	64	1	64/1	2	16	64/16	2	1	8/0.125	0.25	0.06	8/0.002	0.156
BCH33016	128	0.25	64/0.125	1	16	64/8	1	0.25	16/0.06	0.375	0.015	16/0.001	0.188
BCH33022	64	0.25	32/0.125	1	16	32/8	1	0.25	8/0.06	0.375	0.015	8/0.002	0.188
BCH33034	32	1	32/1	2	32	64/16	2	1	8/0.125	0.375	0.015	8/0.004	0.5
BCH33039	32	0.5	32/0.5	2	16	32/16	2	1	16/0.125	0.75	0.03	4/0.002	0.188
BCH33042	64	0.5	32/0.125	0.75	16	32/8	1	1	8/0.03	0.156	0.008	16/0.001	0.375
BCH33056	64	0.5	32/0.25	1	32	64/32	2	0.5	16/0.03	0.313	0.06	32/0.015	0.75
BCH33060	64	0.5	64/0.5	2	16	32/8	0.75	0.5	8/0.125	0.375	0.015	4/0.002	0.188
BCH33061	32	0.5	16/0.125	0.75	16	8/8	0.75	1	8/0.125	0.375	0.06	4/0.002	0.156
BCH33065	128	0.5	64/0.25	1	16	64/8	1	0.5	32/0.06	0.375	0.03	16/0.002	0.188
BCH33072	64	0.5	64/0.5	2	16	64/16	2	1	16/0.125	0.375	0.06	4/0.004	0.125
BCH33073	32	1	32/1	2	32	32/32	2	0.5	8/0.06	0.5	0.03	8/0.008	0.5
BCH33082	16	0.5	16/0.5	2	64	8/32	1	0.25	2/0.03	0.25	0.015	2/0.002	0.25
BCH33100	32	0.25	16/0.125	1	16	16/8	1	1	8/0.125	0.375	0.06	4/0.002	0.156
BCH33105	16	1	16/1	2	16	16/16	2	0.5	4/0.06	0.375	0.03	8/0.015	1
BCH33106	128	1	64/0.5	1	32	128/32	2	1	32/0.06	0.313	0.06	16/0.002	0.156
BCH33115	64	0.25	64/0.25	2	16	64/16	2	0.5	16/0.03	0.313	0.03	16/0.004	0.375
BCH33122	16	1	8/0.125	0.625	16	16/16	2	1	2/0.125	0.25	0.06	8/0.03	1
BCH33126	32	0.5	32/0.5	2	16	16/2	0.625	1	16/0.125	0.625	0.06	16/0.008	0.675
BCH33128	32	1	32/1	2	64	32/64	2	1	16/0.06	0.563	0.008	16/0.002	0.75
BCH33143	64	1	32/0.25	0.75	16	64/16	2	0.5	8/0.015	0.156	0.015	16/0.002	0.375

**Table 2:** MICs of allicin and antifungal drugs tested alone against *M. canis* strains (μg/mL).

**Drug**	**MIC range**	**MIC_**50**_**	**MIC_**90**_**	**GM**
Allicin	16–128	32	128	46.313
Griseofulvin	0.25–1	0.5	1	0.602
Fluconazole	16–64	16	64	22.627
Itraconazole	0.25–1	0.5	1	0.616
Terbinafine	0.004–0.06	0.03	0.06	0.026

The results for each drug combination are listed in Tables 1, 3. Synergism was observed in the following combinations: allicin + itraconazole (86.7%) and allicin + terbinafine (80%) (Tables 1, 3). When synergism was observed, the median reduction in itraconazole was 8-fold (range 4- to 32-fold), while the median reduction in terbinafine was 16-fold (range 4- to 32-fold) (Table 1). Only indifference was observed in the following combinations: allicin + griseofulvin and allicin + fluconazole (Tables 1, 3).

**Table 3:** MIC ranges of drug combinations, FICI ranges and synergistic ratios of tested *M. canis* strains (μg/mL).

**Antifungal combination**	**MIC range**	**FICI range**	**Synergistic ratio**
Allicin + griseofulvin	8–64 (0.125–1)	0.625–2	0
Allicin + fluconazole	8–128 (2–64)	0.625–2	0
Allicin + itraconazole	2–32 (0.015–0.25)	0.156–0.75	86.7%
Allicin + terbinafine	2–32(0.001–0.06)	0.099–1	80%

## Discussion

Although many synthetic antifungal drugs are available for dermatophytosis, the occurrence of resistance or toxic side-effects may lead to the treatment failure. Thus, new therapeutic strategies are necessary. Improvements in the efficacy of synthetic antifungal drugs and reductions in toxicity may be achieved using combination therapy with natural antifungal drugs. Previously studies have demonstrated the effects of herbal essential oils and their synergism with ketoconazole against *Trichophyton* spp (11). In this study, we investigated the *in vitro* antifungal activity of allicin alone and in combination with four antifungal agents (griseofulvin, fluconazole, itraconazole and terbinafine) against clinical isolates of *M. canis*.

Griseofulvin is the first systemic antifungal that was introduced for dermatophytosis. However, its use has been superseded by itraconazole and terbinafine in other types of dermatophytosis (12). Griseofulvin works by disrupting the mitotic spindle structure and inhibiting nucleic acid synthesis (13). Although griseofulvin-resistant *M. canis* strains has been documented in different studies (3, 5), no griseofulvin-resistant strains were detected in our study. In this study, the MIC_50_ and MIC_90_ of griseofulvin were 0.5 and 1 μg/mL, which were consistent with a previous study from Iran (5). Although antagonism was not observed for the combination of allicin and griseofulvin, synergism was not observed either. Their combination in the clinic may not be suitable.

Azoles act on ergosterol biosynthesis by inhibiting 14α-demethylation of lanosterol, leading to altered permeability of the fungal membrane and defective fungal cell wall synthesis (2). In our study, fluconazole had a higher MIC_50_, MIC_90_ and GM than itraconazole, indicating that the latter might be superior for the treatment of tinea capitis. This result was consistent with a previous study from Iran (5). *In vitro* synergistic effects of allicin and fluconazole have been observed against *C. albicans* (8). Interestingly, this combination is indifferent against *M. canis*, which may be due to species differences. In addition, a previous study from Malaysia also demonstrated that allicin in combination with fluconazole showed indifferent interaction against *M. canis* (14). Allicin indeed exhibited synergistic effects in combination with itraconazole against *M. canis* (86.7%).

Terbinafine inhibits the enzyme squalene epoxidase, blocking the synthesis of 2,3-oxidosqualene and thus leading to the accumulation of squalene and the depletion of ergosterol (2). Terbinafine had the lowest MIC_50_ (0.03 μg/mL), MIC_90_ (0.06 μg/mL) and GM (0.026 μg/mL) among the tested drugs against *M. canis* in our study. A previous study from Iran also demonstrated the high *in vitro* antifungal activity of terbinafine against *M. canis* (5). However, it is less useful in the treatment of tinea capitis due to *M. canis*. The MICs of terbinafine against *M. canis* could be higher than the maximum concentration reported in hair, accounting for treatment failure (15). The MICs of terbinafine in combination with allicin significantly declined compared with terbinafine alone, indicating that their combination might enhance the treatment of terbinafine in tinea capitis due to *M. canis*.

The actual antifungal mechanism of allicin is not yet fully understood. It can cause oxidation of glutathione that results in a shift of the cellular redox potential, inducing apoptosis of fungal cells (16). Another transcriptome study revealed that allicin impaired the expression of genes coding for enzymes of amino acid metabolism, iron uptake, the respiratory chain, thiamine metabolism and proteasomal protein degradation of fungal cells (17). The MIC_50_ and MIC_90_ of allicin were 32 and 128 μg/mL in this study. However, a previous study showed a MIC_50_ of 0.098 μg/mL and MIC_90_ of 0.195 μg/mL, which were obviously lower (14). Yamada and Azuma found MICs of *Trichophyton, Epidermophyton*, and *Microsporum* were as low as 0.78–6.2 μg/mL (7). Another previous study reported higher MICs (16–32 μg/mL) of *Trichophyton* to allicin (18), which were consistent with our study. Similar discrepancy had also observed in *C. albicans*. A previous study showed that the MIC range of allicin to tested *C. albicans* strains was 0.025–12.5 μg/mL (19). However, another study showed a very higher MIC range (64–512 μg/mL) of allicin to *C. albicans* strains (8). The discrepancy may be due to the difference of drug resource, tested strains and antifungal susceptibility testing assay. Allicin alone does not have strong antifungal activity, while synergistic antifungal activity of allicin withitraconazole and terbinafine. Allicin may be an adjuvant therapy to traditional itraconazole and terbinafine therapy in tinea capitis caused by *M. canis* to reduce the course of treatment or drug dosage.

In conclusion, we described the antifungal susceptibility of *M. canis* to allicin, fluconazole, itraconazole, terbinafine and griseofulvin and investigated the combined antifungal activity of allicin with antifungal drugs. A major limitation of this work is that resistant strains were not tested. Our study revealed that the combination of allicin with itraconazole and terbinafine may represent an attractive perspective for the development of new management strategies for tinea capitis due to *M. canis*. Further *in vivo* studies are needed to validate our findings.

## Data Availability Statement

The datasets presented in this study can be found in online repositories. The names of the repository/repositories and accession number(s) can be found in the article/supplementary material.

## Author Contributions

YZ and YX contributed to conception and design of the study. YZ and JC recruited patients and collected data. YZ wrote the first draft of the manuscript. All authors contributed to the article and approved the submitted version.

## Conflict of Interest

The authors declare that the research was conducted in the absence of any commercial or financial relationships that could be construed as a potential conflict of interest.

## Publisher's Note

All claims expressed in this article are solely those of the authors and do not necessarily represent those of their affiliated organizations, or those of the publisher, the editors and the reviewers. Any product that may be evaluated in this article, or claim that may be made by its manufacturer, is not guaranteed or endorsed by the publisher.
